# Planting the seed: using research as a tool to revitalize puberty ceremonies in Anishinaabe communities

**DOI:** 10.1108/QRJ-03-2024-0072

**Published:** 2024-10-10

**Authors:** Miigis B. Gonzalez, Alexandra Ziibiins Johnson, Lisa Awan Martin, Naawakwe, Jillian Fish, Lalaine Sevillano, Melissa L. Walls, Lee Obizaan Staples

**Affiliations:** Center for Indigenous Health, Johns Hopkins Bloomberg School of Public Health, Sault Ste. Marie, Michigan, USA; Anishinaabe, Miskwaagamiiwi Zaaga’iganing, Red Lake, Minnesota, USA; Center for Indigenous Health, Johns Hopkins Bloomberg School of Public Health, Sault Ste. Marie, Michigan, USA; Anishinaabe Bawahting, Sault Ste. Marie, Michigan, USA; Anishinaabe, Nagaajiwanaang, Fond du Lac, Minnesota, USA; Macalester College, Saint Paul, Minnesota, USA; School of Social Work, Portland State University, Portland, Oregon, USA; Center for Indigenous Health, Johns Hopkins Bloomberg School of Public Health, Sault Ste. Marie, Michigan, USA; Anishinaabe, Aazhoomog, Hinckley, Minnesota, USA

**Keywords:** Indigenous culture, Elders’ perspective, Indigenous methodology, Indigenous spirituality, Indigenous well-being

## Abstract

**Purpose –:**

The purpose of this work is to honor the wisdoms of Anishinaabe Elders, community and culture by interweaving these teachings with my own (first author) Anishinaabe experiences and a research project. Ceremonies are an important health practice for Anishinaabe people. This project aimed to gain a clearer conceptualization of the protective role of Anishinaabe puberty ceremonies on health in adolescence and across the lifespan.

**Design/methodology/approach –:**

Spiritual offerings guided this project at every stage including inviting Elders and community members into shared spaces of storytelling and teaching elicitation and grounding me as I carefully adopted the use of a western tool (research) in sacred community spaces. Elders were invited to share their experiences and perspectives. Three community members engaged with the interview transcripts on their own before coming together to discuss themes, patterns and insights that arose for them. This group coding discussion constructed the structural foundation of the findings.

**Findings –:**

An Anishinaabe perspective on youth development emerged. Key aspects of this model included a foundation of ceremonial experiences that spiritually prepares a child for adulthood and impending life’s challenges. As one transitions into adulthood, they accept the responsibilities of being caretakers of their families and communities and gain new tools to contribute to Anishinaabe society. Ideally, this society prioritizes Anishinaabe spirituality, language and way of life.

**Originality/value –:**

Frameworks of health, grounded in unique community wisdoms and worldviews, are imperative to repair spiritual and community relationships damaged in a history of colonialism. An Anishinaabe perspective on youth development may shed light on shared Indigenous experiences of cultural restoration and continuity.

## Planting the seed

I (first author) am an Anishinaabe mother, scholar, Anishinaabemowin (Anishinaabe language) learner and advocate for our Anishinaabe way of life and worldview. Anishinaabe (also Ojibwe, Chippewa) are Indigenous to what is now known as Michigan, Wisconsin, Minnesota, North Dakota and central southern Canada. I am excited and hopeful to lead a paper with Indigenous relatives that centers and celebrates our Anishinaabe teachings in an Anishinaabe research agenda (Smith, 2021). Lee “Obizaan” Staples, a notable Anishinaabe first language speaker and an international spiritual advisor, has laid the groundwork for what I know to be true about our spiritual existence as Anishinaabe. Obizaan has guided several language and ceremonial apprentices to carry forward teachings from generations past. Many of these apprentices are working tirelessly to provide for our communities what Obizaan has taught them. I feel immensely blessed that he has accepted me as not merely his student but as his family. My perspective reflects the teachings Obizaan has bestowed onto me.

Linda Tuhiwai Smith’s (1999) seminal work created the momentum for me to deeply engage Anishinaabe teachings in research practices and methodologies and to preserve and share them through story (Archibald, 2008; Kovach, 2021). As Anishinaabe people move in and out of survival, recovery, development and self-determination (Smith, 2021), I move too, as does an Anishinaabe research agenda. The story shared here represents this process of responding to the needs put forth by my Anishinaabe community through research practices that are rooted in Anishinaabe worldviews (Gonzalez et al., 2023). This story is about the important transition between childhood and adulthood, the ceremony Anishinaabe people were given to support that transition and what changes we might see in our holistic community health when these ceremonies are revived.

This story begins in April 2018. My niece was coming out of her 4-day isolation, or what we knew as her puberty ceremony or puberty fast. She was transitioning from a child to a woman in a time period where she would be known as an Oshkiniigikwe (new woman). During the year following, she would learn and practice what it meant to be a woman in Anishinaabe society and learn and practice what she needed to do to “harness her power as a woman” (Brooke “Niiyogaabawiikwe” Gonzalez, personal communication, Oct. 2022). My niece is the eldest of my mother’s grandchildren. Remarkably, she would be the first of our living relatives to be provided with this ceremony, marking the revival of this practice for our family.

I had been working diligently in the four days during her fast to sew her an appliquéd dish bag and wristlet for her feminine products. Oshkiniigikweg are required to carry their own dishes and silverware to protect others from their power during this time. It is thought that women during this time are so powerful that they can hurt or make others sick if they are not careful, especially men and young children who do not harness an equal power. I finished sewing in time and sat back admiring what I was going to present to my niece, in her honor, and in the hopes that she would know how proud of her I was, how much I loved her and how I would always be there to support her in any way I knew how. I am uncertain how a bag could convey all of that, but what I sat there wanting and putting out into the universe was, “I wish I could make one of these for every Oshkiniigikwe in my community, so they too knew how much they were loved and how much pride they should carry forward.” I remember that moment vividly and even in this remembrance, I am filled with joy, love and gratitude for my family, for my community and for all that we have been given as Anishinaabe to be well and to thrive. While I did not continue to think about how I was going to accomplish that specific sewing task, the seed had been planted. It would be years later in reading a call for proposals that I would begin to water this seed. This call for proposals centered the importance of spiritual health. While I did not receive the award, it was just enough nourishment for the seed to sprout. It was at this time that I began to actively cultivate the growth of this idea into a research project, which I am elated to share here in honor of Smith’s (1999) *Decolonizing Methodologies: Research and Indigenous Peoples*.

### Ceremonies as a vital and foundational indigenous health practice

Anishinaabe have several ceremonies conducted across the lifespan. When conducted during one’s childhood, these ceremonies are thought to lay a foundation for living a good life by building spiritual connections to the Manidoog (spiritual helpers) and acknowledging the gifts given to us as Anishinaabe People. By conducting these ceremonies in Anishinaabemowin, we instill deep and sacred beliefs that teach us how to be in relation with ourselves, other beings and the land. This is one thing that continues to distinguish an Anishinaabe research agenda from conventional science – the emphasis on what we hold sacred, spiritual and ceremonial (Smith, 2021).

Ceremony is a necessary and critical component of Indigenous culture and therefore must be considered as we build Indigenous Science that supports culture as prevention (e.g. Hughes et al., 2023; Wilson, 2008). One American Indian (AI) treatment program included a wide variety of ceremonies (e.g. sweat, fasting, naming, rite of passage), and their participants experienced an increased sense of well-being and purpose, knowledge of drug-free lifestyles and connection to positive social networks (Bresette, 2010 as cited in Rowan et al., 2014). Participating in ceremony, learning traditional Native ways, having relationships with Elders and traditional healers and maintaining a cohesive community are considered fundamental healing components among AI community members (Hartmann and Gone, 2012). Ceremony also connects people to spiritual helpers, spiritual messages and spiritual intuition (see “revealed knowledge” in Castellano, 2000), increasing spiritual connectedness and affirming spiritual beliefs (Kulis et al., 2012).

Puberty ceremonies, specifically, are conducted by Indigenous communities across the globe and may act as a foundation for healthy transitions from childhood to adulthood and from adulthood to elderhood (Simard, 2009). For example, within the Indigenous Xhosa-speaking people of South Africa, the Intonjane is a girl’s rite of passage to womanhood ceremony performed at a girl’s first menstruation in which she emerges with new cultural teachings to be applied in her renewed membership to her family and community (Henda, 2021). Amongst Krobo female youth in Ghana, those who participated in Dipo, a puberty rite of passage, were found to have less psychological distress than Krobo female youth who did not (Abbey et al., 2021).

The rituals of puberty ceremonies vary across Indigenous cultures and within cultures as well. Perhaps though, there exist shared values and beliefs that mark this transition as one to be especially nurtured, for the betterment of the young person and their role in Indigenous futures. During the adolescent developmental period when mental health problems proliferate (Walls et al., 2020), these ceremonies instill spiritually grounded values, beliefs and behaviors vital to building personal capacity to live well, gaining personal agency to thrive in the face of adversity, instilling a strong personal and cultural identity and realizing full potential to influence their own life course (Risling Baldy, 2017). Family and community ceremonial involvement inspires a deep sense of belonging and socio-cultural connectedness and expands social support – all of which are critical components of wellness, particularly in Indigenous communities (Ullrich, 2019; Walls et al., 2022).

### Adolescent transitions

Across cultures, adolescence is considered a formative stage of development in which patterns of mental, behavioral, physiological and social growth lay a foundation for health in later life. While most adolescents navigate this phase and emerge with a stronger sense of self, purpose and attachment to others, some adolescents face challenges, demonstrated in high rates of anxiety disorders (Rapee et al., 2019), opioid use (Schuler et al., 2021) and suicide (Leavitt, 2018) among AI youth. Historical trauma has also been associated with injurious health effects including depression and suicidality amongst Indigenous young people (Strickland et al., 2006). Indigenous scholars argue that it is Indigenous traditional practices, cultural and community connectedness, a strong cultural identity and spirituality that may be associated with positive health outcomes and an ability to buffer the deleterious impact of stressors due to colonization and colonialty among young people (Goodkind et al., 2010; Fetter and Thompson, 2023; Fish and Syed, 2018; O’Keefe et al., 2022; Ullrich, 2019). Thus is the motivation to move carefully towards a deeper exploration of one Anishinaabe practice that focuses on this important life phase of a young person, to understand how this ceremony may impact their health in adolescence and across their lifespan.

### Purpose of this study

Puberty ceremonies, a foundational practice of Anishinaabe societies, may be protective against negative health behaviors and outcomes. This study engages Elders and ceremonial leaders in conversations regarding what Anishinaabe puberty rites of passage entail (to protect Indigenous Knowledge, these details will not be shared here) and why maintaining and revitalizing this ceremony is a vital health practice for Anishinaabe communities. Teachings from our Elders will lead future research projects by identifying the healing pathways that may exist between Anishinaabe puberty ceremonies and health in adolescence and across the lifespan.

## The soil

An Anishinaabe Worldview model (Gonzalez et al., 2023) is the guiding framework for this project. Developed in collaboration with Anishinaabe first language speaking Elders, it describes the healing pathway that one may begin when they participate in spiritual practices (i.e., ceremony) by gaining a deeper understanding and belief in spiritual connections, embracing an Anishinaabe identity, and pursuing an Anishinaabe way of life. In the creation of this model, it cannot be overstated the deep-rooted belief and knowledge the Elders carried that confirmed to them: everything that Anishinaabe people were given by the Manidoog is love and good.

During this project, I am also doing the work of caretaking my relationship with research (Kovach, 2021; Lambert, 2014; Smith, 2021). One of our Elders has taught me the importance of feasting and caretaking our tools. She explains that Anishinaabe people have always feasted our tools that help us lead a good life (e.g. ricing knockers, paddles, and hunting gear). Recently, I have experienced her feasting the tools that are used to revitalize our language (e.g., audio recorders, computers, and mic booms). For me, I have taken on research as my relation, as something I have deemed worthy of building a relationship with. Guided by Smith (2021), I am careful to not assume research is harmless; if I let research lead me, it will result in harmful practices. Instead, I merely carry research as one tool that I have been gifted to extend my ability to help my community. With any process that we take to help others (e.g., setting a table, carrying grocery bags, or research), my Elders stress the importance of slowing down, being present, and doing what is necessary to help without harm. When you rush to do things fast, instead of doing things well, that is when we risk breaking a dish or ripping a grocery bag. In research, the fear of “making a mess” is enough to paralyze an Indigenous scholar. Here lies the importance of allowing our asemaa (spiritual currency) to lead. The experiences that I have using my asemaa while working on this project, guide me and weaken the intermittent feelings of unworthiness and doubt. With my asemaa, my worries that research may be tainting something beautiful subsides.

## Cultivating

Integral to an Anishinaabe research agenda, I engaged a Community Research Council (CRC) to co-lead this project and future projects that unfold. Four CRC members were chosen based on their own belief and leadership with the many Anishinaabe life-course ceremonies. Each CRC member is an apprentice to first language speaking Elders and ceremonial leaders as a part of their experiential scholarship. They also wholeheartedly believe that increasing access to Anishinaabe ways benefits individuals, families, and communities. As the PI/first author of this project, I engaged with the CRC, drafted a semi-structured interview guide, conducted each of the interviews, quality checked the transcriptions, facilitated group thematic analysis, and led the writing of this paper.

I also invited each of my community-based coding consultants as authors of this paper: Naawakwe, Alexandra Ziibiins Johnson, and Lisa Awan Martin. As I will describe, it was through their experiential lenses (and wisdoms), that they were willing and capable interpreters of the Elders’ teachings. It was their thoughts and ideas that shaped the structural model of the results.

Additionally, I invited Indigenous academic relatives–Drs. Jillian Fish (Tuscarora), Lalaine Sevillano (Ilocano), and Melissa Walls (Anishinaabe)–to help connect my work with Indigenous scholarship and prepare this paper for publication.

Finally, I include Lee Obizaan Staples as a co-author, collaborator, and leader in this project. Conversations with him all along my journey with this project, including his facilitation of my niece’s ceremony back in 2018, has guided me and my understanding of the importance of this work, and the importance of revitalizing everything we have been given as Anishinaabe. He explains, it is within this practice of *being Anishinaabe* that we are able to experience spiritual fulfillment and live our life’s purpose.

### Elders

Community research partners and Elders decided who would be appropriate to interview, given the topic. Community partners identified participant Elders as people who are knowledgeable about the puberty ceremony, are respected ceremonial leaders and Elders, and have either gone through this ceremony as a young person and/or assist in facilitating this ceremony for young people today. Seven Elders completed the interview and consent process. All participants identified as Anishinaabe and ranged in gender, ages of eldership, and tribal communities.

### Analytic procedure

Interviews were audio recorded and transcribed verbatim. A group of three community coding consultants engaged with the transcripts to identify themes and patterns to answer the research question: What is the role of puberty ceremonies in well-being for young people and across the lifespan? These consultants were chosen based on their high level of engagement and leadership in their community, especially regarding language and cultural revitalization, and they were chosen based on the thoughtfulness and respect that they have shown in prior shared community spaces. I met with each of the coding consultants to explain the project and coding processes. I offered them asemaa and a culturally-relevant gift to thank them and appropriately connect them to the work. Each coding consultant, at their own pace, coded the transcripts (i.e., identified themes, patterns, and significant ideas). After completing individual coding, coding consultants met once in a 90-min meeting to discuss their findings as a group. I was only a listener to this discussion, centering the knowledge and insights of community members (Kovach, 2021). In the week following this discussion, I reread each of the transcripts alongside the notes taken during this group discussion. It was from the perspectives of this group that a structural outline of thematic results effortlessly emerged. I continued to fill in this outline with group thoughts and my own revisiting of the transcripts and my time with the Elders. The final themes and visual representations were shared with community partners, Indigenous colleagues, coding consultants, and Elder participants to ensure that the model was an accurate representation of the Elders’ teachings, and with the hope that the model would be useful in community efforts to revitalize Anishinaabe traditional practices of well-being.

## Sprouting

### Mii iw epenimoyaang “what we rely on.”

“Only the Manidoog know everything.” I preface this results section with this Anishinaabe adage to carefully share what I have learned from our Elders and community partners, without trapping Anishinaabe Knowledge, Perspective, and Worldview in a stagnant or final state. Anishinaabe People and the land that we walk are ever-changing. Therefore, allow me to share what I have learned as an evolving idea, a sprout that will grow, seed and reproduce, or not, in a lifecycle that doesn’t belong to me, that I know very little about.

### All our ceremonies are interconnected

**Figure 1.**
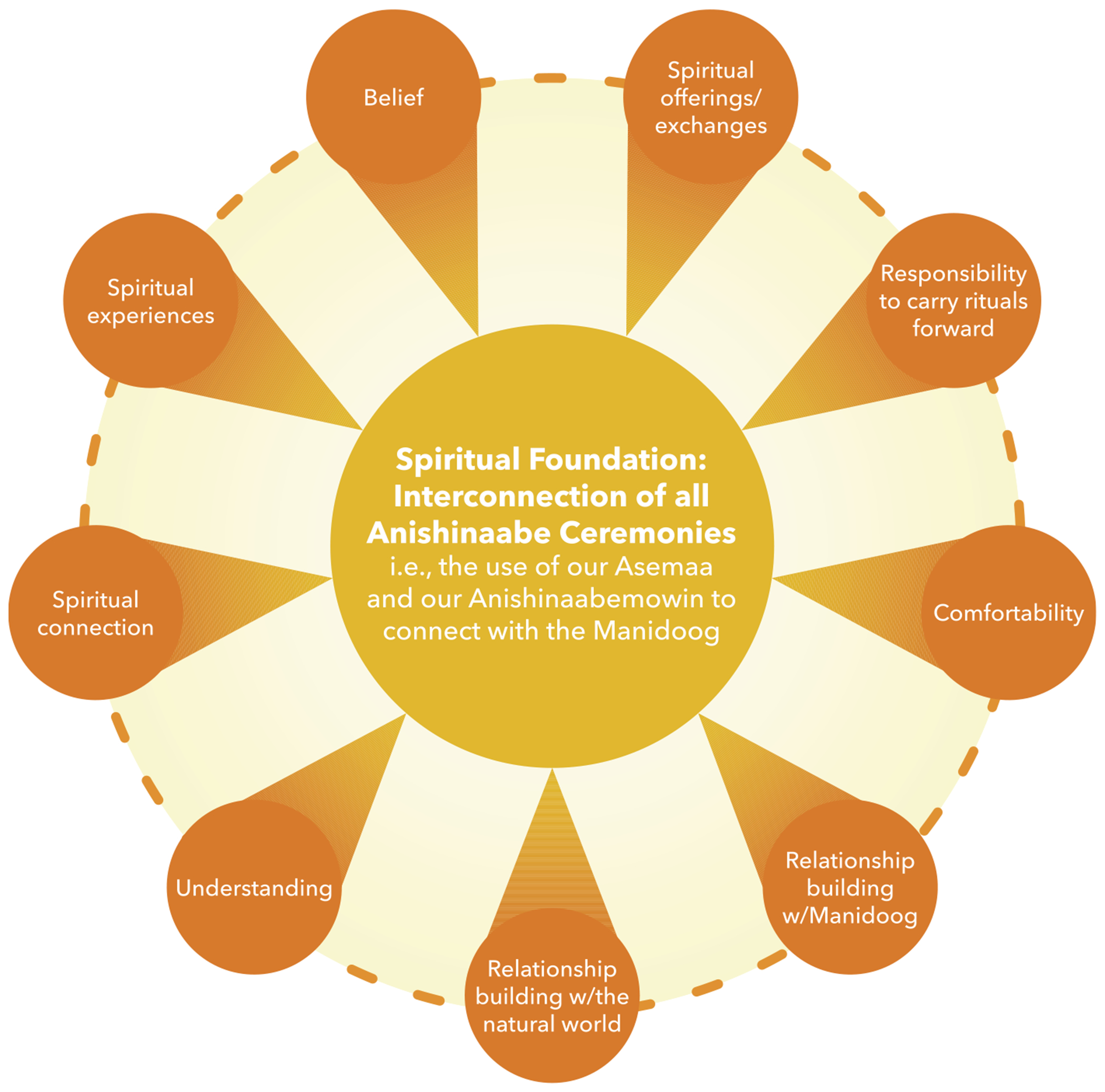
Foundation for ceremonial meaning-making and connection **Source(s):** Figures have been designed and edited by community and JHU partners

Our Elders did not talk about our puberty ceremonies in isolation. They spoke about our puberty ceremonies as being one of many ceremonies. More importantly, there are other ceremonies that one should receive in childhood that creates the necessary foundation for our puberty ceremony to be impactful. Ceremonies are the rituals we have been gifted by the Manidoog to use to give thanks, to receive help, and to act in gracious and responsible ways to protect all life forms. Anishinaabemowin (Anishinaabe language) and asemaa are vital conduits of these rituals, among other things specific to each ceremony. It is all that we have been given as Anishinaabe that feeds the Anishinaabe spirit within the Anishinaabe person. It is for this reason that Obizaan explains for individuals to feel complete, they must seek spiritual fulfillment via pursuing Anishinaabemowin, ceremonies, and Anishinaabe ways of life (see Figure 1).

The Elders spoke about ceremonies as multiplying (i.e., ceremonial experiences contribute to ceremonial experiences). Within these experiences, especially as we participate recurrently, we gain a deeper understanding, a deeper belief, and possibly a deeper spiritual remembrance that “everything that was given to [Anishinaabe] is good.” (JN) Our ceremonies are powerful ways that we, as Anishinaabe people, come together in community with each other, with other spiritual beings that have agreed to help us, to enhance that connection to the Manidoog. The Elders spoke of the help that is accessible to us, when we follow these ways. It is within ceremony, and by using our language and asemaa that we are able to gain the teachings necessary to know how to conduct ourselves and be in right relations with others.

Our Elders were asked if re-introducing our community members to this ceremony, especially adults that did not experience this ceremony during puberty, will be a good thing. They responded: *yes, but* … They noted the importance of preparing our community members who have little to no experience with ceremonies. One Elder said, “I think right now if you were to go out there and poll 10 people, I don’t think they would know what you were talking about … because they’ve never had it themselves.” (JN) Our Elders also spoke of fearbased thinking that exists in our Anishinaabe communities that are remnants of boarding school experiences and the continuation of western society to dismiss contemporary Indigenous injustice. It is this fear-based thinking along with ceremonial inexperience, a result of the same oppression, which produces uncertainty that many of our people may not be ready for a puberty ceremony without a foundation of ceremonial exposure.

I’d tell them, right now we’re living in misery. Some of us are pretty miserable out there, and I think what do we need to do to get out of that misery? I think we need to get back to doing things the Anishinaabe way. Here’s some ceremonies we used to do. I think they deserve a shot … I don’t think the Creator put us here to be miserable. He gave us a lot of things to help us. (JN)

There was no doubt that the Elders believed in the spiritual power and importance that this and other ceremonies play in Anishinaabe well-being. However, these ceremonies must mean something, for us to lean into the help that we can access through these ceremonies. One Elder described our puberty ceremony as an experience to:

… sit with your first family. Your first family isn’t your mom and dad, it’s not your grandma, grandpa, or your children. Our first family is the trees, water, air, the seasons; it’s all that in nature. They’re our Elders. They’ve been here long before we have and they’re gonna stay here long after we’re gone. (SS)

Another Elder similarly described the support available in nature and with the Manidoog in times of grieving*:*

When we talk about grieving at funerals, one of the best places to go is out in the woods, let out your emotions. Lot of Manidoog out there that will help you. A lot of love and support available. A lot of our adults, they don’t realize that they are next door to that support. A lot of our adults live without that support or the knowledge that that exists for them. You would think they would welcome that. But based on that anxiety and that damage that we deal with as a people, we were taught not to have faith. (LS)

Anishinaabe people have access to an immense amount of support, spiritual and otherwise, if we know to seek out that help. When our people have this spiritual understanding, the puberty ceremony supports the important transition we all must make from childhood to adulthood. Our Elders stated that there is no adolescent stage in Anishinaabe society.

For our people, there’s no adolescent stage in a child’s state to adult …. [in] western society, you have the slow adolescent years. And our fast is to go out there, say the boy, before he goes that way out there, and all of his child and childish characteristics, right? So, the obnoxious, loud, raising cane, boy you’ve been. You come back, and you’re considered a man now, which opens the door to the warrior teachings. (TS)

Perhaps then, the completion of the puberty ceremony is the culmination (i.e. graduation ceremony) that marks that the individual is not merely ready for this particular ceremony, but ready for something beyond: To make that transition into adulthood and take on the roles and responsibilities of a contributing adult member of Anishinaabe society.

### Theory of youth development: An Anishinaabe perspective

**Figure 2.**
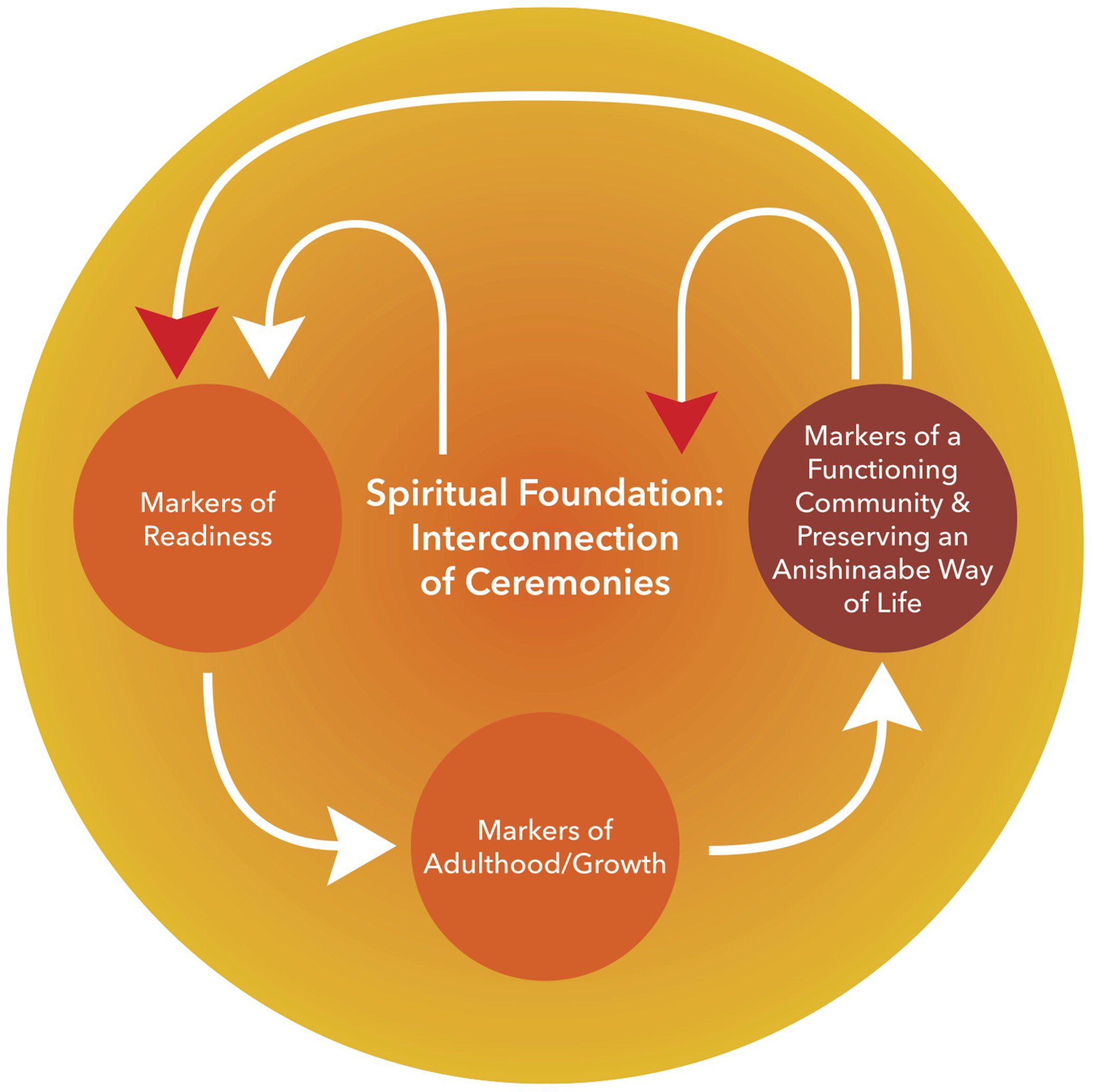
Theory of youth development: an Anishinaabe perspective **Source(s):** Figures have been designed and edited by community and JHU partners

As aforementioned, in Anishinaabe ways, we did not consider an adolescent period. Our developmental milestones emerge within a spiritual framework in the following way:

1. A child is raised within Anishinaabe ways of life and within Anishinaabe ceremonies;
2. When the child shows markers of readiness (physical, mental, emotional, and spiritual), they are offered support for this transition into adulthood; 3. They gain new instructions, via but not exclusively through a puberty ceremony, to fulfill their adult roles and responsibility in community; 4. Their roles and responsibilities strengthen the functionality of the community, which simultaneously contributes back into the continuation of that spiritual foundation (see Figure 2).

**Markers of readiness** into adulthood are not based on age and I am starting to consider that they may not have been based on physiological changes either. These markers of readiness might have once coincided with age and physical changes because we were immersing our children in ceremonies from the time of their birth. Markers of readiness, today, comes from engagement in ceremony and the quest for living an Anishinaabe existence including knowledge we gain outside of ceremony, for example, understanding clan teachings and having a grasp of Anishinaabemowin. When asked to describe the emotional, spiritual, or physical experience of a young person going through this puberty ceremony, one Elder responded:

Depends on their upbringing, what role modeling they had. You know, there’s a whole set of teachings that people are raised with, teaching them to respect everything in life … There’s a Manidoog in that lake, conduct yourself in a respectful manner when you’re out there in that lake. So when kids are brought up with all of that, they respect the ceremonies that we are given as a people. So if they are brought up with all of that, they understand why they are fasting. They’re going in with prior teachings. (LS)

When our young people have gained Anishinaabe teachings and know how to appropriately conduct themselves to respect nature, or to conduct themselves within ceremony, they can transfer those skills to navigate various social contexts. It is these young people that understand they are spiritual beings, loved by the Manidoog, and that each being has a spirit, deserving of kindness and respect. The Elders described that when children are raised in Anishinaabe practices, they carry forward the following markers of readiness:

1. Belief in Anishinaabe ways and teachings
2. Responsibility to Anishinaabe ways and to Anishinaabe community
3. A sense of belonging to these ways and an existing relationship with the Manidoog
4. Understanding of Anishinaabe ways, i.e. understand why we do what we do.
5. Value in Anishinaabe ways
6. Trust in Anishinaabe ways

It is when our people carry these vital foundational principles, that they are ready for adulthood and therefore ready for that ceremony that supports them in their transition to adulthood.

**Markers of adulthood/growth** interweave with a sense of purpose and willingness to contribute to a larger system of life (i.e., family, community, natural world). Showing markers of adulthood or markers of growth means one looks for meaningful ways to contribute. In this stage, they are actively seeking to know themselves at a deeper level and to understand their purpose here on earth. “Creator didn’t just put us here … he put us here for a purpose, and we all have a purpose here.” (JN) Another Elder stated of this time period:

… you start thinking about your future. About your contribution in life and what will be your purpose for existing. Each one of us has been put on this earth for a reason. There is something we are to accomplish while we are here. And it’s about that age when young people start thinking about their future. What is my purpose for existing? What is it I’m supposed to be doing? How will I contribute to my community? What is my role going to be? (LS)

The puberty ceremony, specifically although not exclusively, is a means to understand one’s purpose and earn the gifts that will help us help others. Anishinaabe puberty ceremony requires sacrifice (e.g. fasting, isolation, spiritual offerings). When we make a sacrifice or when we give something of ourselves, we open ourselves up to receiving something in return. These are our gifts, or what the Elders described as the items or tools (tangible and intangible) that we can use to help our community and to live our purpose.

It’s like you’re coming into a new life, maturing. Certainly, guidance in your life, giving you direction in your life. Also, for a lot of them, the Manidoog appear to them, they’re showing you their love. If there are spiritual beings out there that love you, shows you that you’re not alone in this world. They will always be there for support, they are reassuring for a young person, there is love available to them. (LS)

Living our purpose is a spiritual experience, one of joy, contentment, self-acceptance, and confidence. It is when we live our purpose, that we gain the strongest sense of self. Living our purpose is exactly what we need and it is exactly what our community needs from us. One Elder reflected on his ceremony, “… every time I went out I’ve benefited from it a little bit, but the people benefited from it more.” (TS)

As we transition from childhood to adulthood, the Elders talked about the act of putting down or leaving behind old ways and picking up new ways. New ways included making good decisions, using the gifts one has been given, and living a life of purpose. More importantly, the Elders noted that this act of putting down old ways and picking up new ways and the puberty ceremony itself, opens the door to learn and access more. The Elders talked about getting something from this process, but not getting everything. However, by going through this process, you open up a pathway to gain the rest of what you need, throughout your life, when you need it. “The answers might not come out the first time you put out your asemaa, but it’s coming.” (SS) You can also do this ceremony again, no longer thought of as a puberty ceremony, throughout your life if you have something you need, knowledge you hope to gain, or a deeper understanding you hope to achieve.

Making “man decisions” was mentioned by all the male Elders as they reflected on the numerous young men that have come to them seeking guidance to live a better life. Making good decisions meant you had to “let things go” (JN). One Elder talked about a young man, a boy, that couldn’t let things go. He was in and out of jail, going to the bars, and getting into fights. He came out of one of his stints in jail and asked for guidance. This Elder told him “you’re leaving childhood and going into adulthood … when you leave boy to man, now you got to start making man decisions. You can’t be making boy decisions anymore.” He also told that boy, “… I don’t think you’re ready yet.” During that meeting with his Elder, the boy was adamant that he was ready. By the next week, the Elder said, that boy was back in jail. He went to visit him and the boy explained, “A guy disrespected me so I beat the f out of him.” The Elder responded, “There you go, that boy decision. Man decision, you would’ve walked away.” This story elucidates the importance of putting things down, letting things go, and sacrificing, before we are truly ready to pick up something new and ready ourselves for adulthood.

Regarding women, the Elders spoke about the importance of understanding the power women carry. In Anishinaabe ways, women are especially powerful as they are able to create life and have innate instincts in caregiving. The women specific teachings shared by the Elders were meaningful to the female community analysts who read the transcripts. The female analysts spoke about how different that first menses would have been for them had they been given their women teachings at that time. These women analysts stated their Anishinaabe perspective on women as one of empowerment, and as a stark contrast to shame, embarrassment, and fear they experienced as young people.

When asked about non-conforming gender identities, and the differences in their ceremony or teachings, one Elder responded,

The Manidoog are not discriminating. It’s the spirit of who you are, who they identify with … Then I’d tell them that it’s a gift. There’s a reason why you feel you have a spirit gender identified … For a young man to have feminine characteristics, those Manidoog intended for that person to be born that way. And it’s up to [that individual], later on in life, to find out what that’s all about, to have that mixture, that combination in their lives. (LS)

Another responded, “It’s just the way that the Manidoog see them, by their Spirit, that’s how the [ceremonial instructions] came out.” (TS)

Elders talked about the need in our communities today to get back to balance among men and women and among fathers and mothers. Balance requires men and women to be ready for parenthood, because parenting is difficult: “[parenting is] the hardest job in the world, trying to keep those kids on the right track. There’s challenges, they challenge you, which is normal. They’re going to do that. You got to be ready for that.” (JN). This readiness for parenthood is much more than a physiological readiness (i.e. puberty). Parenthood and being an active contributor to one’s community requires mental (e.g. making good decisions), physical (e.g. providing food and shelter), and spiritual (e.g. belief in Anishinaabe ways) readiness. Caring for your loved ones and having family-sufficiency is one of the Anishinaabe markers of adulthood.

Whether it is parenthood, obtaining or losing employment, gaining a new skill, or illness, being an adult means being capable of facing and overcoming the multitude of challenges that come our way. Ceremony, spirituality, living a beautiful Anishinaabe existence does not equate to a perfect, free-of-hardship existence. Our puberty ceremony is difficult. It will test your limits and draw out the strongest parts of you. One Elder stated, “[you] gain perspective on something that other people or you yourself thought was a hardship.” (SS) Living Anishinaabe ways and gaining spiritual experiences through ceremony does however, arm you with the tools and relationships necessary to seek the help you need in those situations. Further, it is within these ceremonies and the relationships you build over your lifetime with the Manidoog, that you receive the gifts that you were destined to use to help your community thrive and to preserve our Anishinaabe way of life into our futures.

**Markers of a functioning community and preserving an Anishinaabe way of life** go hand-in-hand. One Elder quoted his Elder who said:

… we live in two worlds, we live in Anishinaabe world, and we live in chimookomaan (white) world … I think we need to get back to this, we need to get back to being Anishinaabe. And I said, I think once we get back into that, I think everything else will start getting better. (JN)

A functioning community is grounded in Anishinaabe values, beliefs, and unique worldview, which is due to the maintenance of the ceremonial or spiritual rituals that were gifted to the Anishinaabe. One Elder spoke of his Elders’ teachings that, “everything that was given to us is good.” But he went on, “But if we don’t use it, what good is it? If we don’t use it, what do you expect” (JN). The Elders spoke of preserving the rituals that have been passed to them by their Elders. One Elder clarified that we pass on the rituals of what we’ve always done as Anishinaabe in the hopes and with the expectation that within those rituals, the underlying message from the Manidoog and the teachings transfer with them (GJ). Even more important, is preserving Anishinaabemowin that these ceremonies are meant to be delivered within. “… we do our ceremonies that are all done in Ojibwe, because that is the way it was meant to be.” (JN)

Another marker of community functioning is the fulfillment of various roles, e.g. hunters, healers, and helpers. Each adult performs their role, with self-acceptance and understanding, ideally because that role is based in self- and spiritually-identified purpose. These roles are considered deeply tied to clan teachings; clan membership is assigned to us through our parents. One Elder stated that he was unaware of the clan teachings his mother was bestowing onto him, it was just a part of life, and that he learned by watching her and emulating her actions (GJ). Another Elder spoke about the importance of the variety of roles that marks both a functioning ceremony and a functioning community.

… you need everybody. You need your hunters, you need those that cook, you need those that watch the kids, they need those who pick the medicines, you know, and then you know you have your doctors. So, they do see some people running around that didn’t get to have that puberty ceremony and understand what their place is, what their clan is … What does your clan say you should be doing, you know? And so you see our people running around trying to do everything and not feeling good about themselves because they can’t do all of these things and they’re not comfortable just being who they are … Like, we need your singers, or your workers, or your politicians. Back home, only one of our clan systems’ job is to be the politicians, or [Clan name], that’s the worker bee, that’s the people that stack the wood. I do know somebody who’s like that, and they really enjoy that, y’know? When they can accept that’s who they’re supposed to be. (SS)

The Elders described the puberty ceremony in a similar way; that every individual who completes it will receive something different than the next person. You receive exactly what is designed and meant for you. One cannot assume their role or purpose is the exact same as another person’s. How much less envy, jealousy, and hate would exist if we knew not to compare ourselves to any of our fellow Anishinaabe people?

Thus, the puberty ceremony can be one of those ways that roles and purpose are identified, which supports the functionality of community. “We’re all told there is a reason we were put on this earth and there are some who are told through fasting exactly what that is. That’s gotta be amazing.” (LS) It’s our responsibility to take care of our own gifts by appreciating and being grateful for what we have been given, trusting in our intuition, using our gifts to help others, and continuously building spiritual relationships that we can lean on.

Engaging in Anishinaabe ceremonies and contributing to community are ways that Anishinaabe people can break old cycles and create new cycles of well-being and “get back to being Anishinaabe” (JN). One Elder reminisced about a time when Anishinaabe still visited with each other and showed up for each other during important times. She admired her Elder who would urge, “Help each other. Be kind to each other. Play together. Love one another” (MB). This idea of community and family extended beyond Anishinaabe people into the natural world as well. Like the Elder who described this ceremony as sitting with your first family or the Elder who said it is those Manidoog in the woods that will take care of us when we grieve. Those relatives in the natural world are essential members of our families and communities.

Finally, it is also important to note that ceremonies, in all their sacredness and spiritual depth, bring Anishinaabe people together in joy and laughter. As one Elder put it, laughing and teasing is our “love language” (SS). In all seriousness and in the grand task of revitalizing all that has been viciously stolen - we thrive, we love, we tease, and we laugh. Laughing and teasing are markers of a functioning Anishinaabe society and are also necessary components of ceremony. Offering your asemaa and being offered asemaa locks you into that ceremony, and all the energy you emit has an impact on how everything unfolds. This is why Obizaan teaches us not to do things debinaak (carelessly/half-hearted). He urges ceremonial helpers to be present and keep our minds on positive, healing thoughts and energy.

## Budding

An Anishinaabe theory of youth development may be useful as our communities move through this current state of being (i.e., revitalization) into full self-determination (Smith, 2021). Some of the Elders’ viewpoints opposed views upheld by western society. These points of dissension should be examined as many are being upheld deep within the structures of American society and forced blindly on our Anishinaabe youth and therefore on Anishinaabe futures. The Elders talked about “earnings” as our gifts and our roles in society. These oppose western views of earnings (i.e., money), however both are considered vital in our ability to care for ourselves and our families. Obizaan, for example, uses money to spiritually gift others, knowing that money is useful in our modern world. However, he explains any personal sacrifices we make towards spiritual assistance are considerable, because instead of using that money towards something for ourselves, we chose to use that money as a spiritual offering. In addition, the Elders considered that which we earn (i.e., our gifts, our roles), as something that always and without doubt, contributes to Anishinaabe community, society, and futures. Societies exist on a foundation of values, beliefs, histories, and social contexts (Fish et al., 2022). Let us question, then, the training we are providing for *and* requiring of our young people for them to contribute to society, and the society itself. We can contribute to community functioning while simultaneously challenging its underlying values and expectations. We do this by first grounding ourselves and our loved ones in our Indigenous ways of life, which reinforces the foundation by which we proceed.

The Elders’ views of markers of readiness and adulthood challenge common western ideas. In the United States, for example, we are considered adults at around age 18 and able to graduate to “higher education” when we earn a high school diploma. Conversely, an Anishinaabe marker of adulthood is having a deep sense of spiritual support to fulfill one’s purpose, a purpose distinguishable from our peers. Perhaps these different underlying objectives partially affect high rates of AI high school dropout (McFarland et al., 2019) and low rates of AI college matriculation (U.S. Department of Education, 2019 as cited in Tachine and Cabrera, 2021). In addition, the length of substance use treatment programs or jail sentences should mark a readiness to return to society. And yet, there are alarming rates of recidivism and relapse, especially in AI communities (Wodahl and Freng, 2017; Evans et al., 2006). One of our Elders told a story about punishment within his community. Reasonably, his community was enraged about a heavy crime committed. While this Elder understood his community’s response, he reflected, “… maybe that’s something we’ve learned again, learned behavior. I think we still are a kind and forgiving people, not a punishing people” (JN). We must consider how we are preparing our people to be functioning members of society, but also question what societal expectations we aim to uphold. Other conversations with Anishinaabe Elders similarly disputed western ideas of success and well-being (Gonzalez et al., 2023) – a call to deconstruct not merely our measurements of Indigenous culture that may lead to health (O’Keefe et al., 2023; Walls et al., 2016, 2019), but our conceptualizations of Indigenous health, wealth, and community systems.

The Elders urged that we need to “get back to being Anishinaabe,” and “get back to all that we were given as Anishinaabe,” suggesting that “living in two worlds” might not be serving Anishinaabe people as it once did. This perspective is a critical consideration, particularly as two-eyed seeing approaches (Wright et al., 2019) gain prominence and as Indigenous orientations to research evolve. Obizaan explains this spiritual/internal dissonance Anishinaabe people experience when we do not “feed our spirit” with Anishinaabemowin and lifeways (Gonzalez et al., 2023). Cultural theory supports the need for grounding ourselves in our own culture to navigate another (LaFrombroise et al., 1993), and perhaps this should be our focus in the upbringing of our young people. Further, Kimmerer (2013) in *Braiding Sweetgrass*, describes herself as navigating two worlds – Indigenous and scientific. In this way, we have numerous worlds to navigate, e.g. school, work, politics. The autistic community is empowering their people to “unmask” (Radulski, 2022), as they experience devastating outcomes of this learned practice to camouflage within social settings (Pearson and Rose, 2021). Similarly, have we as Indigenous people unknowingly learned to mask, but at the stake of adopting the underlying values and beliefs of a western society? A society that has actively tried to bury our way of life and unique worldviews (Brave Heart and DeBruyn, 1998). As we move into full self-determination, to be unapologetically Anishinaabe (or one’s respective Indigenous identity) is a necessary act of continued resistance. To understand that, as Indigenous scholars, our “academic skills” (Lorde, 1984) or our ability to wield an academic tool, does not define our expertise nor our significant contributions to science. It is the strength in our differences (e.g. our Indigenous Knowledges and Worldviews) that may “bring about genuine change.” (Lorde, 1984). To be political changemakers and defenders of our Indigenous Knowledges, we must challenge, resist, and force our Indigenous voices, and thus our worldviews, into all social contexts (Smith, 2021). Perhaps then these brave everyday practices will make their way into the highest spaces of decision-making, creating an unstoppable move toward health and human equity.

## Flowering

“Miigii, is this for silverware?” She looked amused that I had included a silverware pouch inside her dish bag. This was my second niece to go through her ceremony. She carried a sense of knowing about this process, perhaps something innate, perhaps because she had a shared memory of her older sister’s ceremony. A group of relatives sat around a feast of food and gifts laid out in her honor after completing her four-day fast. We chatted with each other and addressed our Oshkiniigikwe as teachings emerged from our memories. I learned a lot that day from women about their own women’s journey. I yearned for my own ceremony and knew that day would arrive for me too, with my nieces in my lead. My heart smiled as I actively absorbed a memory of her in that moment. It was amazing to witness someone emitting presumably opposing energies: calm yet energetic; content yet beaming; wise but youthful. This is not to say that she always carries these characteristics. She struggles as is natural, sometimes swaying from one energy to the other. It is also not to say that she is not young, unhappy or discontent anymore. But when she is, “she has something to hold onto (minjimaakwii’ind)” (Obizaan). She is in transition, and I could tell even then that she had been prepared.

Smith (2021) points out that Indigenous researchers are tasked with “getting the story right and telling the story well” (p. 282). As an Anishinaabe mother, scholar and advocate, telling the story of Anishinaabe puberty ceremonies means telling my own family’s stories and that of the Elders included here. I am grateful for my experiences as an Anishinaabe woman and scholar, which have been woven together in a path laid out for me by the Manidoog and empowered by my Elders, ancestors and all Indigenous leaders and scholars braving their own journeys. To truly understand something, you have to experience it with your whole being – body, mind and spirit (Cajete, as cited in Kimmerer, 2013). This is not to know it all, as only the Manidoog do, but to trust in and care for what has been given to us as Anishinaabe people: our ways of life, histories, stories, ceremonies and language. If we can pool our energies here, to revive everything that we have been given, what a beautiful life we would be creating for ourselves, our families, our communities and our futures.
